# Genome Comparison of Erythromycin Resistant *Campylobacter* from Turkeys Identifies Hosts and Pathways for Horizontal Spread of *erm*(B) Genes

**DOI:** 10.3389/fmicb.2017.02240

**Published:** 2017-11-15

**Authors:** Diego Florez-Cuadrado, María Ugarte-Ruiz, Guillaume Meric, Alberto Quesada, M. C. Porrero, Ben Pascoe, Jose L. Sáez-Llorente, Gema L. Orozco, Lucas Domínguez, Samuel K. Sheppard

**Affiliations:** ^1^VISAVET Health Surveillance Centre, Universidad Complutense Madrid, Madrid, Spain; ^2^The Milner Centre for Evolution, Department of Biology and Biochemistry, University of Bath, Bath, United Kingdom; ^3^Departamento de Bioquímica, Biología Molecular y Genética, Facultad de Veterinaria, Universidad de Extremadura, Badajoz, Spain; ^4^Subdirección General de Sanidad e Higiene Animal y Trazabilidad, Dirección General de Sanidad de la Producción Agraria, Ministerio de Agricultura y Pesca, Alimentación y Medio Ambiente, Madrid, Spain; ^5^Department of Animal Health, Faculty of Veterinary Medicine, Universidad Complutense Madrid, Madrid, Spain

**Keywords:** *Campylobacter*, erythromycin, *erm*(B), turkey, antimicrobial, transmission

## Abstract

Pathogens in the genus *Campylobacter* are the most common cause of food-borne bacterial gastro-enteritis. Campylobacteriosis, caused principally by *Campylobacter jejuni* and *Campylobacter coli*, is transmitted to humans by food of animal origin, especially poultry. As for many pathogens, antimicrobial resistance in *Campylobacter* is increasing at an alarming rate. Erythromycin prescription is the treatment of choice for clinical cases requiring antimicrobial therapy but this is compromised by mobility of the erythromycin resistance gene *erm*(B) between strains. Here, we evaluate resistance to six antimicrobials in 170 *Campylobacter* isolates (133 *C. coli* and 37 *C. jejuni*) from turkeys. Erythromycin resistant isolates (*n* = 85; 81 *C. coli* and 4 *C. jejuni*) were screened for the presence of the *erm*(B) gene, that has not previously been identified in isolates from turkeys. The genomes of two positive *C. coli* isolates were sequenced and in both isolates the *erm*(B) gene clustered with resistance determinants against aminoglycosides plus tetracycline, including *aad9, aadE, aph(2″)-IIIa, aph(3′)-IIIa*, and *tet*(O) genes. Comparative genomic analysis identified identical *erm*(B) sequences among *Campylobacter* from turkeys, *Streptococcus suis* from pigs and *Enterococcus faecium* and *Clostridium difficile* from humans. This is consistent with multiple horizontal transfer events among different bacterial species colonizing turkeys. This example highlights the potential for dissemination of antimicrobial resistance across bacterial species boundaries which may compromise their effectiveness in antimicrobial therapy.

## Introduction

The World Health Organization (WHO) has recently published a list of bacteria for which new antibiotic therapies are urgently needed, with *Campylobacter* classified as high priority (WHO, 2017). This is of concern as campylobacteriosis is the most commonly notified bacterial foodborne infection in the European Union (European Food Safety Authority [EFSA]/European Centre for Disease Prevention and Control [ECDC], 2016a). The disease is principally caused by *Campylobacter jejuni* and *Campylobacter coli* following the ingestion of contaminated food and drink, with consumption of poultry meat recognized as a major risk factor (Wilson et al., 2008; Whiley et al., 2013). Infection can be associated with extra intestinal pathologies and sequelae such as reactive arthritis or Guillain-Barré syndrome (Nachamkin et al., 1998), but it is usually self-limiting. Treatment of severe infection occasionally requires antimicrobial therapy, often with erythromycin (European Food Safety Authority [EFSA]/European Centre for Disease Prevention and Control [ECDC], 2016b) and to a lesser extent with gentamicin, the later used occasionally when infection becomes systemic (Lehtopolku et al., 2009). Although fluoroquinolones were commonly used in the past, the rising of resistance among *Campylobacter* isolates makes these antibiotics ineffective (Kassem et al., 2016).

Erythromycin inhibits protein synthesis by binding to the ribosome and blocking the exit of the nascent peptide chain (Fyfe et al., 2016). Erythromycin resistance in bacterial isolates from animals and humans is associated with the presence of *erm* genes (Weisblum, 1995). The most widely distributed *erm* gene class is *erm*(B), which encodes an rRNA methylase which produces cross-resistance to macrolides, lincosamides and streptogramins B (MLS_B_ phenotype) (Leclercq, 2002). The *erm*(B) encoded enzyme acts on the 23S rRNA gene by methylating an adenine residue that hinders antibiotic binding-to the ribosome (Weisblum, 1995). The *erm*(B) gene is present in a variety of Gram-positive bacteria, including enterococci, streptococci, and staphylococci (Jensen et al., 1999). The potential for interspecies horizontal gene transfer (HGT) (Conwell et al., 2017) has facilitated the emergence of resistance in multiple species including Gram-negative bacteria of the genera *Bacteroides, Shigella, Escherichia, Klebsiella*, and recently *Campylobacter* (Shoemaker et al., 2001; Soge et al., 2006; Phuc Nguyen et al., 2009; Qin et al., 2014). Resistance in *Campylobacter* has been associated with ribosomal mutations, efflux pumps and the *erm*(B) gene that has been identified in isolates from China and Spain (Qin et al., 2014; Florez-Cuadrado et al., 2016; Fyfe et al., 2016). Recent work has reported high-level erythromycin resistance (MIC ≥ 1024 mg/L) in a *C. coli* isolate carrying *erm*(B)- in a genomic island along with other determinants conferring resistance to aminoglycosides, tetracycline and streptothricin (Florez-Cuadrado et al., 2016). Thus, eight types of *erm*(B)-carrying genomic islands have been differentiated (Qin et al., 2014; Wang et al., 2014; Florez-Cuadrado et al., 2016), all of which share aminoglycoside resistance genes in addition to other determinants, likely leading to co-selection after genetic mobilization (Chapman, 2003).

Improved understanding of the distribution of resistance genes within bacterial species in different host niches, and the mobility of these genes between populations, could be important for identifying source and sink populations. In the case of *Campylobacter*, the *erm*(B) gene has been identified in *C. coli* isolates from chicken, ducks, swine, and humans and from *C. jejuni* isolated from chicken (Qin et al., 2014; Wang et al., 2014; Deng et al., 2015) but other host species may be relevant. Turkeys are among the top 10 farmed animals in Europe and the United States with an estimated 323 million birds reared anually (FAOSTAT, 2017). While studies have shown that turkeys are an important host species harboring large numbers of *C. jejuni* and *C. coli*, the resistance status of these strains is not well-characterized. This has lead to the inclusion of this animal species in international surveillance programs to evaluate the levels of antibiotic resistance. In this study we carried out combined molecular microbiology and whole genome sequencing approaches to evaluate the presence of *erm*(B) and it’s genetic background in *Campylobacter* isolates obtained from turkeys sampled in Spain. The comparison with *erm*(B) sequences from other host species might allow further description of the microevolutionary events associated with the acquisition of this antibiotic resistance genes in *Campylobacter*.

## Materials and Methods

### Strains and Growth Conditions

*Campylobacter* isolates were recovered in 2014 (*n* = 170; 133 *C. coli* and 37 *C. jejuni*) from turkey samples obtained in the framework of the European Antimicrobial Resistance Surveillance program (DC 652/2013) in Spain (European Comission, 2013). Samples were collected at the largest turkey slaughterhouses in Spain located in different regions within the country. Each *Campylobacter* isolate represented a single farm and they were obtained by culturing pooled feces from turkeys (117 pooled samples: 10 animals per pool, 1170 individual fecal samples analyzed). Each pooled sample was cultured on *Campylobacter* blood-free selective medium (CCDA) (Oxoid). Inoculated media were incubated at 42°C for 48 h under microaerobic conditions with a commercial gas-generating system (atmosphere generator system, Oxoid). Suspected colonies were subcultured onto blood agar (BioMérieux) at 37°C for 48 h. All strains were identified by conventional multiplex PCR of the genus *Campylobacter* that allows the differentiation between *C. coli* and *C. jejuni* with specific primers, as described previously (Ugarte-Ruiz et al., 2012).

### Antimicrobial Susceptibility Testing

Broth microdilution methods were performed to determine the antimicrobial susceptibility of the *Campylobacter* isolates [minimum inhibitory concentrations (MICs)]. The following antimicrobials were tested: tetracycline, ciprofloxacin, nalidixic acid, erythromycin, streptomycin, and gentamicin. Isolates were grown on blood agar plates (bioMérieux) and incubated for 48 h at 37°C under microaerophilic conditions. Growth from these cultures was suspended in sterilized water and adjusted at 0.5 McFarland. Fifty microliters of these inocula were added to 11 mL of cation-adjusted Mueller-Hinton broth (TREK Diagnostics Systems), and supplemented with 600 μL of lysed horse blood prepared in house from defibrinated horse blood (Oxoid). EUCAMP2 microdilution plates (TREK Diagnostics Systems) were inoculated and incubated under microaerophilic conditions at 37°C for 48 h. *C. jejuni* strain ATCC 33560 was used as a control for antimicrobial susceptibility test. Following the commission decision 2013/652/UE, the epidemiological cut-offs values considered were those described by EUCAST (EUCAST, 2017). *Campylobacter* isolates resistant to erythromycin (MICs: >8 mg/L to *C. coli* and >4 mg/L to *C. jejuni*) were selected to evaluate the presence of *erm*(B) gene.

### Identification of *erm*(B) Gene and Whole-Genome Sequencing

The RNA methylase gene *erm*(B) was identified by PCR as described previously (Chen et al., 2007). Amplicons were detected by gel electrophoresis using 2% agarose gels containing 10 mg/ml SYBR Safe DNA gel stain (Invitrogen) for 40 min at 100 mA. DNA fractions were sequenced and compared using Sanger sequencing and MEGA software (version 5.05) respectively (Tamura et al., 2011; Heather and Chain, 2016). *erm*(B)-positive *Campylobacter* isolates were selected for whole genome sequencing. For DNA extraction, *Campylobacter* isolates were grown on blood agar plates (48 h at 42°C under microaerophilic conditions) and DNA was extracted using a QiAmp DNA mini kit (Qiagen). DNA was quantified using a Nanodrop spectrophotometer before sequencing. High-throughput genome sequencing was performed using a benchtop MiSeq sequencer (Illumina), and the short read paired-end data was assembled using the *de novo* assembly algorithm, SPAdes (Bankevich et al., 2012). Genome sequences were archived in the web-accessible Bacterial Isolate Genome Sequence Database: BIGSdb (Jolley and Maiden, 2010), which included functionality for identifying MLST profiles based on the pubMLST database^1^. Allelic diversity was evaluated using a gene-by-gene approach for genome alignment and comparison with the BLAST algorithm as previously described (Sheppard et al., 2012).

## Results

### Antimicrobial Susceptibility Testing

A total of 170 *Campylobacter* isolates (133 *C. coli* and 37 *C. jejuni*) were tested for susceptibility to six antimicrobials. Antimicrobial resistance profiles (**Table 1**) and MIC distributions were recorded (Supplementary Table S1). The highest proportion of antimicrobial resistance was to tetracycline (168/170; 98.8%) followed by nalidixic acid/ciprofloxacin (164/170; 96.4%), erythromycin (85/170; 50%), streptomycin (82/170; 48.2%), and finally gentamicin (13/170; 7.6%). Considering *C. coli* and *C. jejuni* separately, higher prevalence of resistance was observed in *C. coli* for erythromycin, streptomycin, and gentamicin (Fisher’s exact test: *p* < 0.001). Based upon The European Food Safety Authority (EFSA) criteria for quantifying multi-drug resistance (MDR; resistance to at least three classes of antimicrobials tested), seven MDR profiles were recorded for *Campylobacter* isolates (**Table 1**) (European Food Safety Authority [EFSA]/European Centre for Disease Prevention and Control [ECDC], 2017). Seventy-nine *C. coli* isolates (59.4%) and three *C. jejuni* isolates (8.5%) showed resistance to ciprofloxacin and erythromycin (treatment used against campylobacteriosis before onset of resistance and the current treatment against this bacteria). *Campylobacter* isolates resistant to erythromycin (**Table 1**) were analyzed for the presence of RNA methylase gene *erm*(B).

**Table 1:** Drug resistance profiles found among 170 *Campylobacter* isolates from turkeys.

Resistance Profile^1^	*C. coli No. of isolates (%)*	*C. jejuni No. of isolates (%)*
CTESG	9 (6.76)	0 (0)
CTES	46 (34.58)	1 (2.70)
CTSG	3 (2.25)	0 (0)
CTEG	1 (0.75)	0 (0)
CTE	23 (17.29)	2 (5.40)
CTS	20 (15.03)	1 (2.70)
TES	2 (1.5)	0 (0)
CT	29 (21.80)	28 (75.67)
TE	0 (0)	1 (2.70)
C	0 (0)	1 (2.70)
T	0 (0)	2 (5.40)
Susceptible	0 (0)	1 (2.70)
Total	133 (100.00)	37 (100.00)

^1^*C, ciprofloxacin; T, tetracycline; E, erythromycin; S, streptomycin; G, gentamicin. Combined resistance to nalidixic acid and ciprofloxacin were observed in both *Campylobacter* species, therefore resistance is described to ciprofloxacin only*.

### *erm*(B)-Carrying *Campylobacter* Isolates from Turkeys

**FIGURE 1:**
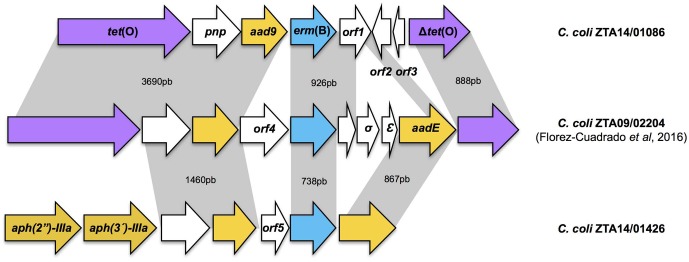
Comparative genetic organization of *erm*(B)-carrying genomic islands from *Campylobacter coli* ZTA14/01086 (this study), *C. coli* ZTA 14/01426 (this study) and previously identified *C. coli* ZTA09/02204 (17). Antimicrobial resistance genes are colored as follows: *erm*(B) gene (blue); aminoglycoside resistance genes (yellow); the tetracycline resistance gene *tet*(O) (purple). Genes with other predicted functions or encoding hypothetical proteins are shown in white. Gray shading indicates regions sharing 98% DNA identity.

Among the 85 erythromycin-resistant isolates, two (2.4%) carried the *erm*(B) gene: *C. coli* ZTA14/01086 and *C. coli* ZTA14/01426. The *C. coli* ZTA14/01086 is resistant to ciprofloxacin/nalidixic acid, tetracycline, erythromycin and streptomycin, whereas *C. coli* ZTA14/01426 shares the same MDR pattern and gentamicin resistance. The erythromycin-resistance MICs were 256 mg/L for *C. coli* ZTA14/01086 and >1024 mg/L for *C. coli* ZTA14/01426. Whole genome sequencing revealed that the *erm*(B) gene in *C. coli* ZTA14/01086 was located in a cluster along with other resistance genes, including *tet*(O) and *aad9* which confer resistance to tetracycline and spectinomycin respectively (**Figure 1**; GenBank accession number: MF134831). The genome of *C. coli* ZTA14/01426 isolate contained the *erm*(B) gene in a cluster with aminoglycoside resistance genes *aph(2″)-IIIa, aph(3′)-IIIa*, and *aadE* (**Figure 1**; GenBank accession number: MF134832). Both genomic islands were compared with the *erm*(B)-carrying genomic island from *C. coli* ZTA09/02204, identified in Spain (GenBank accession number: KT953380) (**Figure 1** and Supplementary Table S2). Other than these resistance determinants, analysis of 23S rRNA and ribosomal protein (L4 and L22) genes from genomes of the *erm*(B) positive strains from turkeys, revealed the absence of ribosomal mutations previously related with erythromycin resistance (Gibreel et al., 2005).

### *erm*(B) Allelic Variation among Bacterial Genera, Hosts, and Countries

**FIGURE 2:**
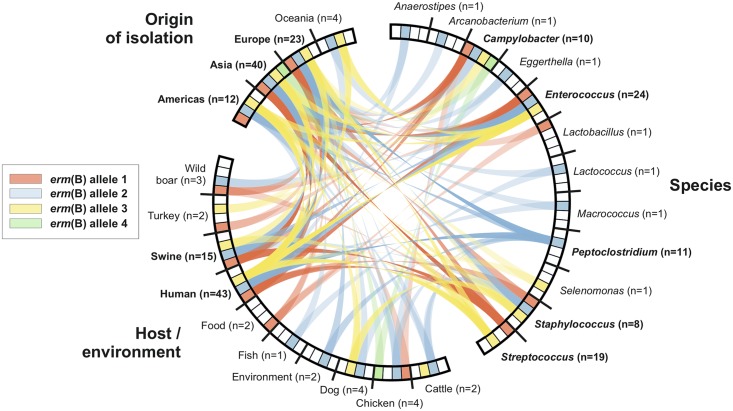
Host and geographical distribution of the *erm*(B) alleles identified in *Campylobacter* in this study among other bacterial genera. Allele 1 belongs to *C. coli* ZTA14/01426 (MF134832), allele 2 belongs to *C. coli* ZTA09/02204 (KT953380), allele 3 belongs to *C. coli* ZTA14/01086 (MF134831) and allele 4 belongs to *C. jejuni* C179b (KF864551). *erm*(B) homologs were identified in GenBank using BLAST with a coverage and similarity of 100%. Accession numbers of each sequence is given in Supplementary Table S3. Sequences without host data and geographical location have not been included. Exact identity of *erm*(B) alleles between species, host/environment and origin of isolation is represented by a connection (width proportional to relative prevalence).

Comparison of sequence homology can provide information about the horizontal transfer of resistance genes, including *erm*(B), among bacterial species. The nucleotide sequences of 10 *erm*(B) genes present in *Campylobacter* were compared, two from this study and eight of Chinese and Spanish origin. Using the first *erm*(B) sequence described in *Campylobacter* as reference (*C. coli* ZC113; GenBank accession number: KC575115), four alleles have been identified in the *erm*(B) sequences from *Campylobacter* (Supplementary Table S3). Allele 1 was the most common (7/10) and was used as reference. Alleles 2 and 3 were present in only one *C. coli* isolate each from Spain, with SNPs A299G (Asn-100-Ser) and A353G (His-118-Arg), respectively. Allele 4 was found in a *C. jejuni* isolate from China and is characterized by the synonymous SNP C726T. Bacterial genera, hosts and origins with *erm*(B) sequence identical to the four alleles observed in *Campylobacter* were identified in the NCBI database, compiled (Supplementary Table S3) and compared (**Figure 2**). The *erm*(B) alleles detected in *Campylobacter* had been previously identified mainly in *Enterococcus* and *Staphylococcus* isolated from humans and pigs in Asia and Europe. Allele 1 of *erm*(B) is represented in 31 sequences from eight bacterial species, including *Streptococcus suis* (11/31; 35.5%), *Enterococcus faecium* (7/31; 22.5%), and *C. coli* (7/31; 22,5%). *S. suis* isolates were mainly isolated in China (7/11; 63.6%) and all were from swine hosts. Sequences of *E. faecium* were mainly from Japan (4/7; 57.1%) and all were of human origin. The majority of *erm*(B) allele 1 sequences from *Campylobacter* were of Chinese origin (6/7; 85.7%) and sampled from humans, swine, and chickens. Allele 2 of *erm*(B) was detected in 16 bacterial species mainly *Clostridium difficile* (10/39; 25.6%) and *E. faecium* (7/39; 17.9%), from European and United States people and pigs of Chinese origin, respectively. Allele 2 of *erm*(B) is slightly less common in NCBI database than allele 1, being the only one identified in six bacterial genomes, mainly *E. faecium* (10/22; 45.4%) sampled from humans in the United States, Australia, Japan and South Korea, whereas allele 4 of *erm*(B) was not identified in any other sequence available in public databases.

## Discussion

Definitive characterization of erythromycin resistance in bacterial pathogens is an important objective defined by the EFSA and the European Centre for Disease Prevention and Control (ECDC) (European Food Safety Authority [EFSA]/European Centre for Disease Prevention and Control [ECDC], 2017). To date, the *erm*(B) gene has been identified in *Campylobacter* isolates from swine, chickens, ducks and humans from China and in one broiler sample from Spain (Wang et al., 2014; Florez-Cuadrado et al., 2016). Monitoring antimicrobial resistance in *Campylobacter* isolated from turkeys has been mandatory since 2014 in European countries where the production of turkey meat exceeds 10,000 tons per year (2016). The occurrence of erythromycin resistant *C. coli* isolates from turkeys in Germany, Romania and Spain had risen to 43.3% in 2014 (2016), compared to previous surveys where lower frequencies were detected for poultry (14.5%) and swine (20.7%) (European Food Safety Authority [EFSA]/European Centre for Disease Prevention and Control [ECDC], 2015, 2016b). Since *Campylobacter* infections are related to consumption of food from animal origin, these levels of resistance could potentially produce therapeutic failure of antibiotic treatment for campylobacteriosis in humans.

Increased antimicrobial resistance among *Campylobacter* populations is a consequence of the widespread acquisition of antimicrobial resistance and clonal expansion of resistant lineages (Wimalarathna et al., 2013). *Campylobacter* can acquire DNA, including antimicrobial resistance genes, from relatively distantly related lineages through HGT, involving the replacement of homologous sequences, or the acquisition of mobile genetic elements (MGEs). There is evidence that plasmid acquisition mediates *Campylobacter* resistance to tetracycline, chloramphenicol and aminoglycosides (Courvalin et al., 1978; Taylor et al., 1987; Wang and Taylor, 1990) but in some cases, these resistances might be conferred by polymorphism of chromosomal sequences. This is also the case in *Campylobacter* for resistance to fluoroquinolones and macrolides, mediated by mutations in *gyrA* or 23S rDNA sequences, respectively (Engberg et al., 2001). Mutations that confer antimicrobial resistance can occur independently in multiple lineages but can also spread by natural transformation followed by homologous recombination, leading to the dissemination of antimicrobial resistance among bacteria that share an ecological niche (Meric et al., 2015).

The increasing number of studies identifying *erm*(B) genes in *Campylobacter* suggest that horizontal transfer is related to macrolide resistance in *Campylobacter* (Qin et al., 2014; Wang et al., 2014; Deng et al., 2015; Florez-Cuadrado et al., 2016; European Food Safety Authority [EFSA]/European Centre for Disease Prevention and Control [ECDC], 2017). High levels of resistance among *C. coli* isolates have been previously reported in isolates from livestock (Thakur and Gebreyes, 2005), potentially reflecting a selective advantage in these niches and clonal expansion of resistance lineages. This study shows the location of *erm*(B) gene in a genomic island along with other antimicrobial resistance genes including *tet*(O), encoding resistance to tetracycline and *aph(3′)-IIIa, aacA-aphD, aad9* and *aadE*, conferring resistance to different aminoglycosides (Qin et al., 2014; Wang et al., 2014; Florez-Cuadrado et al., 2016), a clustering that implies the possibility of co-selection as an evolutionary mechanism (Chapman, 2003). In addition, linked *erm*(B) and *aph(2”)-IIIa* genes detected in the genome of *C. coli* ZTA14/01426 isolate, could be transferred to virulent strains limiting seriously the effectiveness of the two main choices for treatment of severe campylobacteriosis, erythromycin and gentamicin (Lehtopolku et al., 2009).

Although the presence of the *erm*(B) gene was evaluated on a total of 85 *Campylobacter* isolates, the scope of the genomic comparison was limited to only two *C. coli* because they were positive for the erythromycin resistance gene. Despite the limited number of genomes sequenced, the *erm*(B)-carrying genomic islands identified in *Campylobacter* isolated from Spain show genetic differences in comparison with ones from China. Thus, the genomic islands identified in Spain do not correspond to any of the six types of genomic islands identified in *C. coli* from China (Qin et al., 2014; Wang et al., 2014; Florez-Cuadrado et al., 2016). All of the *erm*(B)-carrying genomic islands posses aminoglycoside resistance genes but the gentamicin resistance gene *aph(2”)-IIIa* is present only in Spanish isolates. Resistance to gentamicin was represented in the Chinese *erm*(B)-carrying sequences with the presence of the *aacA-aphD* gene and was identified in *C. coli* isolates from humans, poultry, and swine (Wang et al., 2014).

Identical antibiotic resistance gene nucleotide sequences have been detected in both Gram-positive and Gram-negative bacteria. This is consistent with HGT facilitating the spread of resistance genes between distantly related species (Trieu-Cuot et al., 1985; Courvalin, 1994). Gram-positive bacteria including *Enterococcus, Streptococcus*, and *Staphylococcus* are widely known to harbor various resistance genes (Zilhao et al., 1988; Pinto-Alphandary et al., 1990) and recently, tetracycline and aminoglycoside resistance genes from Gram-positive bacteria have been identified in Gram-negative bacteria including *Escherichia coli* or *Klebsiella pneumoniae* (Arthur et al., 1987; Brisson-Noel et al., 1988) and *Campylobacter* (Lambert et al., 1985; Zilhao et al., 1988; Pinto-Alphandary et al., 1990). Four *erm*(B) alleles were identified in *Campylobacter* in this study (Supplementary Table S3) and comparison of these alleles with those from other bacteria provides useful information about possible routes of transmission (**Figure 2**). First, 100% nucleotide identity with *erm*(B) genes from *Enterococcus, Streptococcus, Peptoclostridium, Anaerostipes, Arcanobacterium, Eggerthella, Lactobacillus, Lactococcus, Macrococcus*, and *Selenomonas* suggests horizontal acquisition from Gram-positive bacteria, as previously described (Qin et al., 2014; Wang et al., 2014; Florez-Cuadrado et al., 2016). Second, the *erm*(B) alleles identified in this study have been previously identified mainly in human and porcine bacterial pathogens of Asian and European origin (**Figure 2**). The pig and human pathogen *S. suis* is considered a reservoir of antibiotic resistance genes (Palmieri et al., 2011) and transferable genetic elements carrying the *erm*(B), *tet*(O) and aminoglycoside resistance genes have previously been reported in *S. suis* isolates (Palmieri et al., 2012). The use of combined antibiotic therapies on swine farms potentially selects for bacterial isolates carrying resistance to different antibiotics (Zhu et al., 2013).

Allele 2 of the *erm*(B) gene has been identified in more bacterial genera than the other alleles in this study (**Figure 2**). All bacterial genera where *erm*(B) allele 2 has been identified belong to the Firmicutes, with two exceptions that belong to the Actinobacteria. These bacterial genera are present in the gastrointestinal tract and species like *Enterococcus, Streptococcus*, and *Staphylococcus* share the niche with *Campylobacter* (Zilhao et al., 1988). The transfer of antibiotic resistance genes in the human colon between bacteria of different genera has been reported (Huddleston, 2014) and it is possible that the antibiotics supplied in both animals and humans could facilitate the selection of strains carrying these multiresistant genomic islands and favor their dispersion. Identifying *erm*(B) genes and MDR genomic islands in *Campylobacter* isolates from turkeys, adds another component to the already extensive network of bacterial genera and hosts involved in the possible dispersion of critical antibiotic resistance genes and MGEs. This study highlights the need to sequence a greater number of *Campylobacter* genomes with the objective of evaluating the impact of genomic islands on the dispersion of antimicrobial resistance genes in this genus.

## Author Contributions

Contributions to the conception: DF-C, AQ, MP, LD, and SS; design of the work: DF-C, MU-R, GM, BP, JS-L, and GO; analysis or interpretation of data: DF-C, MU-R, GM, and BP; drafting the work or revising it critically: DF-C, MU-R, GM, AQ, MP, BP, JS-L, GO, LD, and SS; final approval of the version published: DF-C, MU-R, GM, AQ, MP, BP, JS-L, GO, LD, and SS.

## Conflict of Interest Statement

The authors declare that the research was conducted in the absence of any commercial or financial relationships that could be construed as a potential conflict of interest.

## Footnotes

1 https://pubmlst.org/campylobacter; accessed May 9, 2017
