# Supplementary material for: Genome Comparison of Erythromycin Resistant Campylobacter from Turkeys Identifies Hosts and Pathways for Horizontal Spread of erm(B) Genes
Source: Front Microbiol. 2017 Nov 15;8:2240. doi: 10.3389/fmicb.2017.02240 (PMC5695097; doi:10.3389/fmicb.2017.02240)
Supplement: Supplementary file 1 [file Data_Sheet_1.PDF]

S1 File. MIC distribution for *Campylobacter* isoltes

Antimicrobial Agents	Species	MIC Range (mg/L)	ECOFF (mg/L)[1]	Isolates per MIC (mg/L)												Resistant Isolates (%)
				0.12	0.25	0.5	1	2	4	8	16	32	64	128	>128	
Tetracycline	*C. coli*	0.5-64	2								1	4	19	109		133 (100)
	C. jejuni		1			2		1		3	1	8	13	9		35 (95)
Ciprofloxacin	*C. coli*	0.12-16	0.5			1			21	42	50	19				132 (99)
	C. jejuni		0.5	4		1	1	3	17	11						32 (88)
Nalidixic Acid	*C. coli*	1-64	16							1		17	72	43		132 (99)
	C. jejuni		16						3	1		15	16	2		33 (89)
Erythromycin	*C. coli*	1-128	8				49	2		1	2	5	5	17	52	81 (61)
	C. jejuni		4				33			1			1	2		4 (11)
Streptomycin	*C. coli*	0.25-16	4		2	12	30	9		1	5	74				80 (60)
	C. jejuni		4		16	16	3					2				2 (5)
Gentamicin	*C. coli*	0.12-16	2	11	79	29	1				6	7				13 (10)
	C. jejuni		2	23	11	3										0 (0)

[1]EUCAST: *C.coli* and *C. jejuni* data from the EUCAST MIC distribution website last accessed 2 June 2017

S2 File. Genetic annotation of the genomic islands identified in *Campylobacte*r isolates from this study.

Strain	Gene or ORF	Protein	Homologous Protein[a]	Amino Acid Identity (%)	Annotation/Blast hit
C. coli ZTA14/01086	*tet*(O)	639	639	639/639(100)	TetM/TetW/TetO/TetS family tetracycline resistance ribosomal protection protein [*Campylobacter*] / WP_021137831
	pnp	256	256	255/256(99)	Phosphorylase [*Campylobacter coli*] / WP_057033214
	aad9	259	160	160/160(100)	Aminoglycoside nucleotidyltransferase ANT9 [Campylobacter]/ WP_057031337
			68	57/68(84)	
	erm(B)	245	245	243/245(99)	23S rRNA (adenine(2058)-N(6))-methyltransferase Erm(B) [*Campylobacter coli*] / WP_060794049
	orf1	85	288	42/42(100)	Aminoglycoside nucleotidyltransferase ANT6 [Firmicutes] / WP_006426509
	orf2	108	108	105/108(97)	Hypothetical protein [*Campylobacter coli*] / WP_072238494
	orf3	81	81	81/81(100)	Hypothetical protein [Bacteroidales] / WP_032585129
	tet(O)	639	295	294/295(99)	TetM/TetW/TetO/TetS family tetracycline resistance ribosomal protection protein [*Campylobacter coli*] / WP_002812495
C. coli ZTA14/01426	*aph*(2")-IIIa	303	306	301/303(99)	Aminoglycoside O-phosphotransferase APH(2")-IIIa [*Campylobacter coli*] / WP_057037356
	aph(3")-IIIa	264	264	264/264(100)	Aminoglycoside O-phosphotransferase APH(3')-IIIa [Bacteria] / WP_001096887
	pnp	256	256	254/256(99)	phosphorylase [*Campylobacter coli*] / WP_057033214
	aad9	218	218	218/218(100)	Streptomycin 3''-adenylyltransferase [*Campylobacter coli* CVM N29716] / ERF99003
	orf4	139	139	138/139(99)	ATPase associated with various cellular activities family protein [*Clostridioides difficile*] / WP_021397753
	erm(B)	245	245	244/245(99)	23S rRNA (adenine(2058)-N(6))-methyltransferase Erm(B) [*Campylobacter coli*] / WP_060794049
	ant6	288	288	287/288(99)	Aminoglycoside nucleotidyltransferase ANT6 [Firmicutes] / WP_006426509

[a]Homologous protein from the organism listed in the annotation/Blast hit column

S3 File. Host and geographical distribution of the *erm*(B) alleles identified.

Allele	Specie	Country	Host	GeneBank ID
1	*Campylobacter coli* (reference)	China	Swine	KC575115
	Campylobacter coli[1]	Spain	Turkey	This study
	Campylobacter coli	China	Swine	KJ610808
	Campylobacter coli	China	Human	KC876752
	Campylobacter coli	China	Human	KC876751
	Campylobacter coli	China	Human	KC876750
	Campylobacter coli	China	Chicken	KC876748
	Enterococcus faecium	Japan	Human	NG_047804
	Enterococcus faecium	China	Wild boar	KJ645709
	Enterococcus faecium	Japan	Human	JN899585
	Enterococcus faecium	Japan	Human	JN899583
	Enterococcus faecium	Japan	Human	JN899582
	Enterococcus faecium		Human	HM565169
	Enterococcus hirae	China	Food	CP015517
	Lactobacillus plantarum	France	Food	FJ374272
	Staphylococcus aureus	Taiwan	Human	LC102479
	Staphylococcus aureus	Taiwan	Human	LC125352
	Streptococcus pyogenes	Italy	Human	FN677480
	Streptococcus suis	China	Wild boar	CP017142
	Streptococcus suis	China	Swine	KT336321
	Streptococcus suis	China	Swine	CP015557
	Streptococcus suis	Canada	Swine	CP011419
	Streptococcus suis	China	Swine	CP003922
	Streptococcus suis	China	Swine	CP002644
	Streptococcus suis	China	Swine	CP002640
	Streptococcus suis	China	Swine	CP002465
	Streptococcus suis	Italy	Human	FN997652
	Streptococcus suis	Italy	Human	FN677479
	Streptococcus suis	Vietnam	Human	FM252032
2 (A299G)	*Anaerostipes hadrus*	China	Human	CP012098
	Arcanobacterium pyogenes	United States		AY334073
	Campylobacter coli[2]	Spain	Chicken	KT953380
	Peptoclostridium difficile	Germany	Human	HG475346
	Peptoclostridium difficile	Germany	Human	HF678446
	Peptoclostridium difficile	Italy	Human	HF678445
	Peptoclostridium difficile	United States	Human	FN668944
	Peptoclostridium difficile	Ireland	Human	FN668375
	Peptoclostridium difficile	United States	Cattle	FN665654
	Peptoclostridium difficile	Italy	Human	AM072511
	Peptoclostridium difficile	Italy	Human	AJ968665
	Clostridium perfringens	Canada	Chicken	JQ655732
	Eggerthella spp.	Japan	Human	AP012211

	Enterococcus faecalis	Taiwan	Human	AB563188
	Enterococcus faecium	Australia	Human	LT598667
	Enterococcus faecium	China	Swine	KX156279
	Enterococcus faecium	China	Swine	KX156278
	Enterococcus faecium	Germany	Human	CP011830
	Enterococcus faecium	China	Wild boar	KJ645709
	Enterococcus faecium	China	Swine	KF421157
	Enterococcus faecium	Japan	Human	JN899587
	Lactococcus garvieae	Japan	Fish	AB290882
	Macrococcus caseolyticus	Japan	Human	AP009486
	Peptoclostridium difficile	Spain	Environment	LK933416
	Peptoclostridium difficile	Spain	Environment	LK932404
	Staphylococcus aureus	Germany	Cattle	FN806789
	Staphylococcus aureus	China	Swine	JX560992
	Staphylococcus intermedius	Switzerland	Dog	AF239773
3 (A353G)	*Campylobacter coli*[3]	Spain	Turkey	This study
	Enterococcus faecium	South Korea	Human	CP019210
	Enterococcus faecium	United States	Human	CP018072
	Enterococcus faecium	United States	Human	CP012468
	Enterococcus faecium	Australia	Human	LT603681
	Enterococcus faecium	United States	Human	CP013996
	Enterococcus faecium	Australia	Human	KR066794
	Enterococcus faecium	Australia	Human	CP006623
	Enterococcus faecium	Japan	Human	JN899586
	Enterococcus faecium	Japan	Human	JN899584
	Selenomonas spp.	United States	Human	CP014240
	Staphylococcus pseudointermedius	United States	Dog	CP015626
	Staphylococcus pseudointermedius	United States	Dog	CP016073
	Staphylococcus pseudointermedius	Spain	Dog	JF909978
	Streptococcus pneumoniae	France	Human	HG799498
	Streptococcus pneumoniae	Germany	Human	HG799489
	Streptococcus pneumoniae	Canada	Human	CP002925
	Streptococcus pneumoniae	Italy	Human	FN667862
	Streptococcus pneumoniae	Taiwan	Human	CP001033
	Streptococcus suis	Canada	Swine	CP012731
	Streptococcus suis	China	Swine	CP002644
4 (C726T)	*Campylobacter jejuni*	China	Chicken	KF864551

[1]*C. coli* ZTA14/01426, [2]*C. coli* ZTA09/02204, [3]*C. coli* ZTA14/01086
